# Hospitalization, death, and probable reinfection in Peruvian healthcare workers infected with SARS-CoV-2: a national retrospective cohort study

**DOI:** 10.1186/s12960-022-00787-0

**Published:** 2022-12-22

**Authors:** Willy Ramos, Nadia Guerrero, Edwin Omar Napanga-Saldaña, José Medina, Manuel Loayza, Jhony A. De La Cruz-Vargas, María Vargas, Luis Ordóñez, Yovanna Seclén-Ubillús, Carlos Álvarez-Antonio, Juan Arrasco

**Affiliations:** 1 Centro Nacional de Epidemiología, Prevención y Control de Enfermedades, Ministerio de Salud, Calle Daniel Olaechea 199 Jesús María, Lima, 15072 Peru; 2 Instituto de Investigaciones en Ciencias Biomédicas (INICIB), Facultad de Medicina, Universidad Ricardo Palma, Av. Alfredo Benavides 5440, Santiago de Surco, Lima, 15039 Peru; 3 Unidad de Post Grado, Facultad de Medicina, Universidad Nacional Mayor de San Marcos, Lima, Peru; 4 Centro de Prevención y Control de Enfermedades, Dirección Regional de Salud de Loreto, Iquitos, Peru

**Keywords:** COVID-19, Healthcare workers, Death, Reinfection

## Abstract

**Background:**

Peru has some of the worst outcomes worldwide as a result of the SARS-CoV-2 pandemic; it is presumed that this has also affected healthcare workers. This study aimed to establish whether occupation and other non-occupational variables were risk factors for possible reinfection, hospitalization, and mortality from COVID-19 in cohorts of Peruvian healthcare workers infected with SARS-CoV-2.

**Methods:**

Retrospective cohort study. Healthcare workers who presented SARS-CoV-2 infection between March 1, 2020, and August 6, 2021, were included. Occupational cohorts were reconstructed from the following sources of information: National Epidemiological Surveillance System, molecular tests (NETLAB), results of serology and antigen tests (SICOVID-19), National Registry of Health Personnel (INFORHUS), and National Information System of Deaths (SINADEF). The incidence of probable reinfection, hospitalization, and death from COVID-19 was obtained in the cohorts of technicians and health assistants, nursing staff, midwives, dentists, doctors, and other healthcare workers. We evaluated whether the occupation and other non-occupational variables were risk factors for probable reinfection, hospitalization, and death from COVID-19 using log-binomial and probit binomial models, obtaining the adjusted relative risk (RR_AJ_).

**Results:**

90,398 healthcare workers were included in the study. Most cases were seen in technicians and health assistants (38.6%), and nursing staff (25.6%). 8.1% required hospitalization, 1.7% died from COVID-19, and 1.8% had probable reinfection. A similar incidence of probable reinfection was found in the six cohorts (1.7–1.9%). Doctors had a higher incidence of hospitalization (13.2%) and death (2.6%); however, they were also those who presented greater susceptibility linked to non-occupational variables (age and comorbidities). The multivariate analysis found that doctors (RR_AJ_ = 1.720; CI 95: 1.569–1.886) had a higher risk of hospitalization and that the occupation of technician and health assistant was the only one that constituted a risk factor for mortality from COVID-19 (RR_AJ_ = 1.256; 95% CI: 1.043–1.512).

**Conclusions:**

Peruvian technicians and health assistants would have a higher risk of death from COVID-19 than other healthcare workers, while doctors have a higher incidence of death probably linked to the high frequency of non-occupational risk factors. Doctors present a higher risk of hospitalization independent of comorbidities and age; likewise, all occupations show a similar risk of probable reinfection.

## Background

Since the onset of the COVID-19 pandemic, healthcare workers have been identified as one of the groups at most risk due to their direct exposure to SARS CoV-2 in the course of providing healthcare [1–6]. Thus, the first reports made in China and later in European countries [7–11] evidenced the rapid and exponential transmission in healthcare personnel. As of May 7, 2020, health personnel accounted for 22% of COVID-19 cases in Spain [9]. In the region of the Americas, according to the Pan American Health Organization, as of August 20, 2021, 1,792,212 cases and 10,302 deaths from COVID-19 were reported in healthcare workers [6].

The increased risk of SARS-CoV-2 infection in healthcare workers is explained by their greater exposure to patients with COVID-19, to the procedures that generate aerosol, to the prolonged use of personal protective equipment (masks, gloves, gown, eye protection), or to the lack of any protective equipment [12, 13]. For this reason, the World Health Organization, as part of their public health strategies to safeguard their health and guarantee the continuity of healthcare, prioritized the vaccination of healthcare workers [14].

Although healthcare workers have a much higher risk of SARS-CoV-2 infection and adverse outcomes than the general population, this risk may vary by occupational group and analyzed result (reinfection, hospitalization, and death). Likewise, the risk granted by healthcare is added to that derived from individual risk factors such as age, sex, and diagnosis of comorbidities, among others [2, 4, 5, 7].

Peru has some of the worst outcomes worldwide as a result of the SARS-CoV-2 pandemic, having reached the highest cumulative global mortality rate per hundred thousand inhabitants since the first wave, a phenomenon that continues to this day [15]. It is to be assumed that these outcomes have also been transferred to healthcare workers, during the time of the greatest pandemic activity, with a great incidence of infections, reinfections, hospitalizations and deaths [16–18]. To reduce the transmission of SARS-CoV-2, the Peruvian government established various measures such as remote work for tasks that could be carried out from home and for people belonging to risk groups, which included healthcare workers [19].

In order to quantify the risk of adverse outcomes associated with SARS-CoV-2 infection in Peruvian healthcare workers, it is crucial to differentiate between occupational and non-occupational risk factors. The purpose of this study was to establish whether occupation and other non-occupational variables were risk factors for possible reinfection, hospitalization, and mortality from COVID-19 in cohorts of Peruvian healthcare professionals infected with SARS-CoV-2.

## Methods

This was a national retrospective cohort study made from secondary sources (databases). The studied population consisted of healthcare workers who presented SARS-CoV-2 infection between March 1, 2020 and August 6, 2021. All healthcare workers who presented SARS-CoV-2 infection were included in the study, whether they were symptomatic or asymptomatic, as long as they were positive or reactive to RT-PCR, antigen, or serology testing; in the case of serology reagents, we took into account those that presented a positive result or were reactive to IgM or IgM/IgG (recent infection). Healthcare workers older than 70 years and those with inconsistent records were excluded from the study. Sampling was not carried out; we worked with all healthcare workers who met the inclusion and exclusion criteria because they were accessible through secondary sources.

The cohort of healthcare workers infected with SARS-CoV-2 was reconstructed from the following sources of information: National Epidemiological Surveillance System (NOTI COVID-19), molecular test results (NETLAB), serology results, and antigen (SICOVID-19), National Registry of Health Personnel (INFORHUS) and National Deaths Information System (SINADEF) uniting them to form a database.

From the resulting database, the incidence of probable reinfection by SARS-CoV-2 as well as hospitalization and death by COVID-19 was obtained in six occupational cohorts: technicians and health assistants; nursing staff; midwives; dentists; doctors; and other healthcare workers. This last category grouped together biologists, health career interns, veterinarians, nutritionists, chemical pharmacists, medical technologists, and psychologists. We assessed if these occupation cohorts constituted a risk factor for probable reinfection by SARS-CoV-2, hospitalization, and death by COVID-19. The following occupational and non-occupational variables were also evaluated:

- Probable SARS-CoV-2 reinfection: Work institution, work region where the hospital is located, direct contact with COVID-19 cases in the work environment, age, sex, and diagnosis of comorbidities. Within the work institution, the hospitals of the Ministry of Health and regional governments (MINSA/GORE), Social Security of Peru (EsSalud), National Police and Armed Forces (PNP/FF. AA) and private establishments were considered.
- Hospitalization for COVID-19: Work region, year of hospitalization, sex, age, diagnosis of comorbidities and likely reinfection.
- Death by COVID-19: Work region, year of hospitalization, age, sex, and diagnosis of comorbidities.

A health worker was considered to have probable reinfection if he or she presented more than one positive or reactive laboratory result separated by at least 3 months [20]. Death due to COVID-19 was defined as death occurring as a consequence of the natural history or clinical course of the disease (without recovery period) and must meet at least one of the following criteria [20]:

- Virological: Death in a health worker with clinical disease who dies within 60 days after a molecular (RT-PCR) or reactive antigen test for SARS-CoV-2.
- Serologic: Death in an infected health worker with clinical disease who dies within 60 days of a positive IgM or IgM/IgG serologic test for SARS-CoV-2.
- Radiological: Death in an infected healthcare worker with clinical disease who presents a radiological, tomographic, or nuclear magnetic resonance image compatible with COVID-19 pneumonia.
- Epidemiological link: Death in a health worker with pneumonia that has an epidemiological link with a case of COVID-19. Death in a healthcare worker with pneumonia who registered in the data base to have had an epidemiological link with a COVID-19 case.
- Epidemiological investigation: Death in a health worker suspected of COVID-19 that is verified by an epidemiological investigation. Death of a healthcare worker with suspected COVID-19 who required an epidemiological investigation by the epidemiology personnel, who concluded that the most probable cause of death was COVID-19.
- Clinical criteria: Death in a suspected case of COVID-19 with a clinical picture compatible with the disease.
- Death certificate: Death in a health worker who has a death certificate in which the diagnosis of COVID-19 is presented as the cause of death.

Descriptive statistics were performed based on the obtained frequencies, percentages, measures of central tendency, and dispersion. In addition, bivariate statistics were performed with Pearson's Chi-square test, which was used to compare proportions. To assess the risk of developing adverse outcomes of SARS-CoV-2 infection, multivariate statistics were performed with a log-binomial model (probable reinfection and death) and probit binomial model (hospitalization), obtaining the adjusted relative risk (RR_AJ_) for other covariates or potentially confounding variables as well as their confidence intervals. In this model, probable reinfection, hospitalization, and death were considered dependent variables, and occupational and non-occupational variables (older adults, male gender, and presence of comorbidities) were considered independent variables. The calculations were made with a confidence level of 95%.

## Results

### Healthcare workers characteristics

The study included 90,398 healthcare workers who presented with SARS-CoV-2 infection during the study period. Most cases were seen in technicians and health assistants and in nursing staff.

Results indicated a higher frequency of female cases and a total of 8.9% comorbidity in the entire group. They worked in MINSA/GORE establishments in the department of Lima. 17.2% of the cases reported having direct contact with cases of COVID-19 in their work environment. 85.9% presented with symptoms of the disease. The most used test to confirm the sickness was serology and RT-PCR. This is shown in Table 1.

**Table 1:** Non-occupational, occupational, clinical, and laboratory characteristics of healthcare workers infected with SARS-CoV-2 in Peru

Description	Cases	%
Non-occupational		
Sex		
Male	26,785	29.6
Female	63,613	70.4
Age group		
18–29 (young)	17,544	19.4
30–65 (adult)	70,935	78.5
65–70 (older adult)	1919	2.1
Comorbidity		
Heart disease	2445	2.7
Obesity	2144	2.4
Bronchial asthma	1914	2.1
Diabetes	1535	1.7
Lung disease	384	0.4
Cancer	230	0.3
Renal disease	193	0.2
Liver disease	180	0.2
Neurological disease	141	0.2
Immunodeficiency	83	0.1
Any comorbidity	8082	8.9
Occupational		
Occupation		
Technician and health assistant	34,853	38.6
Nursing staff	23,182	25.6
Doctor	16,117	17.8
Midwife	6256	6.9
Dentist	2162	2.4
Other health professionals	7828	8.7
Labor status		
Appointed worker	44,471	49.2
Contracted worker	33,945	37.6
Resident doctor	1547	1.7
Rural and Marginal	1978	2.2
Urban Health Service		
Intern	862	1.0
Not specified	7595	8.4
Work institution		
MINSA/GORE	64,087	70.9
ESSALUD	16,006	17.7
PNP / Armed Forces	3459	3.8
Others	4761	5.3
Private establishments	2085	2.3
Work region		
Lima	33,631	37.2
Rest of the Coast	19,847	22.0
Mountain range	25,691	28.4
Jungle	11,229	12.4
Direct contact with known COVID-19 cases in the workplace		
Yes	15,526	17.2
No	74,872	82.8
Clinics and laboratories		
Clinical presentation		
Symptomatic	77,643	85.9
Asymptomatic	12,755	14.1
Positive or reactive diagnostic test		
RT-PCR	43,150	47.8
Antigen test	13,424	14.8
Serology	48,280	53.4

### Adverse outcomes of SARS-CoV-2 infection

Our analysis also found that 8.1% of those infected required hospitalization and 1.7% died from COVID-19, while 1.8% presented with probable reinfection. A similar incidence of probable reinfection was observed in the six occupational cohorts (between 1.7% and 1.9%), while it was the doctors who presented the highest incidence of hospitalization (13.2%) and death (2.6%) (Table 2). Doctors also presented greater susceptibility not linked to occupational variables. In this sense, 3.7% of doctors were older adults compared to 2.1% of technicians and health assistants, 1.6% of nursing staff, 1.0% of midwives, 1.5% of dentists and 1.5% of the rest of healthcare workers (Pearson's Chi-square test; *p* < 0.001). Furthermore, 13.1% of doctors had comorbidities compared to 8.6% of nursing staff, 8.5% of dentists 8.1% of midwives, 7.8% of technicians and health assistants, and 7.7% of the rest of healthcare workers (Pearson's Chi-square test; *p* < 0.001).

**Table 2:** Adverse outcomes of SARS-CoV-2 infection in healthcare workers in Peru

Adverse outcomes	# of cases	Incidence (%)
Probable reinfection		
Technician and health assistant	629	1.8
Nursing staff	417	1.8
Midwife	119	1.9
Dentist	39	1.8
Doctor	269	1.7
Other health professionals	125	1.9
Total	1598	1.8
Hospitalization		
Technician and health assistant	2587	7.4
Nursing staff	1598	6.9
Midwife	328	5.2
Dentist	152	7.0
Doctor	2124	13.2
Other health professionals	511	6.5
Total	7300	8.1
Death		
Technician and health assistant	663	1.9
Nursing staff	224	1.0
Midwife	55	0.9
Dentist	40	1.9
Doctor	418	2.6
Other health professionals	121	1.5
Total	1521	1.7

The risk of probable reinfection was similar across the different occupational cohorts of healthcare workers. Regardless of occupation, a higher risk of probable reinfection was observed in those who worked outside of Lima. It was also higher in those who had direct contact with COVID-19 cases in their work environment and in those who had some comorbidity (Table 3).

**Table 3:** Multivariate analysis of occupation and other possible risk factors for probable SARS-CoV-2 reinfection in healthcare workers

Risk factors	RR_AJ_**	CI 95%
Occupational		
Occupational cohorts		
Technician and health assistant	1.055	0.872–1.277
Nursing staff	1.084	0.888–1.324
Midwife	1.018	0.792–1.309
Dentist	1.118	0.783–1.596
Doctor	1.059	0.856–1.309
Other health professionals*	1	
Work institution		
MINSA/GORE	1.388	0.919–2.098
ESSALUD	1.091	0.711–1.675
PNP / Armed Forces	1.176	0.716–1.934
Others	0.808	0.492–1.328
Private establishments*	1	
Work region		
Jungle	2.665	2.319–3.063
Mountain range	1.349	1.170–1.554
Rest of the Coast	1.273	1.111–1.459
Lima*	1	
Direct contact with COVID-19 cases in the workplace		
Yes	3.209	2.749–3.747
No*	1	
Non-occupational		
Gender		
Male	0.907	0.807–1.019
Female*	1	
Older adult		
Yes	0.853	0.584–1.245
No*	1	
Comorbidities		
Yes	1.344	1.156–1.563
No*	1	

*Reference category

**Log-binomial model

The multivariate analysis shows that doctors (RR_AJ_ = 1.271) and to a lesser extent, nursing staff (RR_AJ_ = 1.143) and technicians and health assistants (RR_AJ_ = 1.043) presented a higher risk of hospitalization. Regardless of the occupation, a higher risk was observed in those who worked in Lima and other parts of the coast. It was also a higher rate in those with some comorbidities and the older adults, the latter being the ones with the highest risk (RR_AJ_ = 2.273). The risk was also statistically significant, albeit mild, greater in those hospitalized in 2020 and those that presented a probable reinfection. This is shown in Table 4.

**Table 4:** Multivariate analysis of occupation and other possible risk factors for hospitalization for COVID-19 in healthcare workers

Risk factors	RR_AJ_**	CI 95%
Occupational		
Occupational cohorts		
Technician and health assistant	1.120	1.067–1.176
Nursing staff	1.143	1.085–1.203
Midwife	1.043	0.973–1.118
Dentist	0.987	0.900–1.083
Doctor	1.271	1.208–1.338
Other health professionals*	1	
Work region		
Lima	1.206	1.156–1.257
Rest of the Coast	1.122	1.072–1.124
Mountain range	1.058	1.011–1.106
Jungle*	1	
Year of hospitalization		
2020	1.130	1.099–1.162
2021*	1	
Non-occupational		
Older adult		
Yes	2.273	2.137–2.419
No*	1	
Gender		
Male	1.479	1.440–1.520
Female*	1	
Comorbidities		
Yes	1.742	1.682–1.804
No*	1	
Likely reinfection		
Yes	1.105	1.004–1.216
No*	1	

*Reference category

**Probit binomial model

**Fig. 1:**
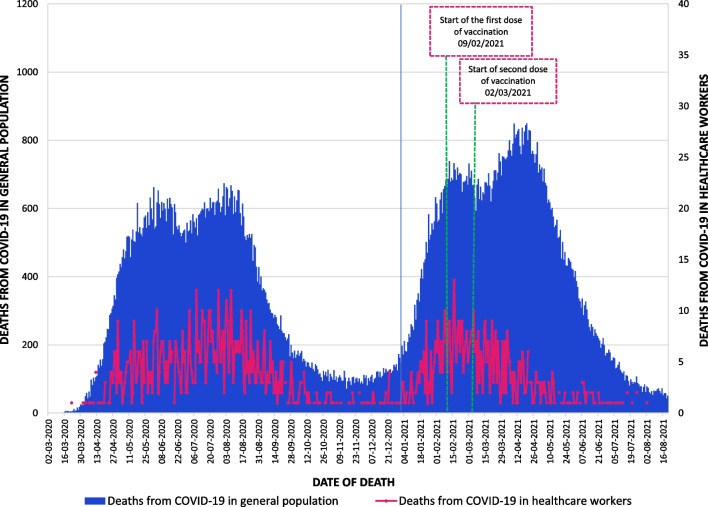
Trend of COVID-19 deaths in healthcare workers compared to the general Peruvian population

The trend of deaths from COVID-19 in healthcare workers, compared to the trend in the general Peruvian population, is shown in Fig. 1. After controlling for occupational variables, it was found that the occupation of technician and health assistant was the only one that constituted a risk factor for mortality from COVID-19 (RR_AJ_ = 1.256; 95% CI: 1.043–1.512). There was also a higher risk of death for those who worked in Lima, those who died in 2021 (during the second pandemic wave), for those who had some comorbidity, males and for older adults. The latter are the ones with the highest risk (Table 5).

**Table 5:** Multivariate analysis of occupation and other possible risk factors for death from COVID-19 in healthcare workers

Risk factors	RR_AJ_	CI 95%
Occupational		
Occupational cohorts		
Technician and health assistant	1.256	1.043–1.512
Nursing staff	0.853	0.685–1.061
Midwife	0.867	0.632–1.189
Doctor	0.940	0.773–1.142
Dentist	1.063	0.775–1.498
Other health professionals*	1	
Work region		
Lima	1.444	1.230–1.694
Rest of the Coast	1.173	0.982–1.401
Mountain range	0.980	0.820–1.174
Jungle*	1	
Year of death		
2021	1.279	1.162–1.407
2020*	1	
Non-occupational		
Gender		
Male	2.720	2.440–3.032
Female*	1	
Older adult		
Yes	8.896	7.939–9.969
No*	1	
Comorbidity		
Yes	2.537	2.275–2.829
No*	1	

*Reference category

**Log-binomial model

## Discussion

This study shows that the risk of hospitalization and death due to COVID-19 varies among occupational cohorts of Peruvian healthcare workers. It is also noted that non-occupational factors have a significant impact on the probability of SARS-CoV-2 infection-related complications.

Death from COVID-19 represents the main adverse outcome of SARS-CoV-2 infection. The case fatality rate (CFR) in healthcare workers in Peru infected by SARS-CoV-2 found in our study was 1.7%, which is higher than that CFR reported by Gholami [21], who found mortality of 1.5% in a meta-analysis of 28 studies which grouped healthcare workers from five countries (China, USA, Netherlands, Italy, Germany, and Spain). A second meta-analysis by Gómez-Ochoa [22] that included 97 studies carried out in healthcare workers in the USA, countries of Asia and Europe, found that mortality from COVID-19 in infected workers was 0.5%, notably lower than that reported for Peruvian healthcare workers. Our results were also higher than mortality from COVID-19 obtained by Bandyopadhyay [23] in a systematic review that included studies up to May 2020 where global mortality among healthcare workers was 0.92%. However, the regional analysis shows similar results since, in Americas, 2.0% of healthcare workers with COVID-19 had died, which represents an intermediate situation among regions with low mortality such as Europe (0.6%) and other with greater mortality such as Eastern Mediterranean (5.7%) and Southeast Asia (3.1%). The countries that reported the highest number of deaths from COVID-19 in Bandyopadhyay’s study were Italy, USA, United Kingdom, Russia, Iran, Ecuador, Indonesia, Mexico, Spain, Philippines, China, Turkey and France. Gholami, Gomez-Ochoa, as well as Bandyopadhyay, included studies that reported the CFR of healthcare workers without carrying out comparisons with the general public.

It should be emphasized that the meta-analysis conducted by Gholami included 119,883 healthcare workers infected with SARS-CoV-2, whereas the meta-analysis conducted by Gómez-Ochoa contained 96,813, and the Peruvian health worker cohort alone had 90,672 infected healthcare workers. This shows the great impact caused by the pandemic among Peruvian healthcare workers in absolute terms. Some reasons for this high risk among Peruvian healthcare workers may be the high workload, continuous exposure, and lack of personal protective equipment (especially at the beginning of the pandemic), but also because of the informal nature of work and the worsening labor conditions observed in many countries before the pandemic, particularly in low- and middle-income countries [16, 24–26].

The results of our research show that the cohort with the highest risk of death from COVID-19 was that of technicians and health assistants, who had a 25.6% higher risk of dying than other healthcare workers. One possible explanation is that the cohort of technicians and health assistants includes technicians and auxiliaries in nursing, laboratory, dental, pharmacy, nutrition, radiology, rehabilitation, and physical therapy. This occupational cohort, particularly nursing technicians, who collaborate with patient care in consulting rooms, emergencies, hospitalization (including feeding, cleaning, mobility, and patient oxygen administration, among others), as well as radiology and laboratory technicians, have close contact with patients while taking X-rays (radiology technicians) or while drawing blood samples and/or manipulation of biological samples for their analysis (laboratory technicians) [27]. This leads to a higher viral load exposure and would explain their greater overall risk [28–30]. The bibliographic review does not find studies with the category of technicians and health assistants like that defined in Peru, but an approximation is found in the cohort of Mexican healthcare workers that finds a higher risk of death in medical assistants, laboratory technicians, pharmacy, and radiology staff [31].

The case of the doctors is particular because they present an occupational risk of dying similar to other healthcare workers; however, they are the ones with the highest unadjusted mortality (2.6%). This is consistent with the results of a systematic review [23] that found that the group with the highest mortality among infected healthcare workers was doctors (6.0%). One possible explanation for this phenomenon is that in Peru, the doctors cohort is the one with the highest proportion of older adults and comorbidities compared to the other cohorts of healthcare workers, which could explain their higher mortality from COVID-19, regardless of their occupational risk. Another possible explanation is that doctors have performed diagnostic tests less frequently in the presence of mild disease and more frequently in the presence of moderate and severe disease, which could have biased the results towards greater lethality [32].

It is observed that non-occupational risk factors lead to a higher risk of death from COVID-19 than occupational factors, the main one being older adults; thus, older adults have about nine times the risk of dying than those under 65 years of age. Male gender, as well as the presence of comorbidities, are risk factors for death from COVID-19. This agrees with other studies carried out on healthcare workers, such as the one carried out by Ferland in 9 European countries [33] and the one by Robles-Pérez in Mexico [30]. The main comorbidities identified were cardiovascular disease, obesity, bronchial asthma, and diabetes mellitus, representing 8.9% of the total healthcare workers infected in this study. These values are similar to those found in the meta-analysis by Gómez-Ochoa, who found that the prevalence of comorbidities was 7% (95% CI: 4–10%) [22].

The trend of COVID-19 mortality among healthcare professionals is comparable to that of the general Peruvian population; however, this correlation breaks down following the introduction of immunization. Thus, the trend in mortality reduced following vaccination, whereas the tendency in the general population was to climb until reaching its peak during the second wave of the pandemic. This confirms the results of Escobar-Agreda [34], who found a higher survival rate in Peruvian healthcare workers in 2021 after the start of vaccination. This would show the effectiveness of vaccination since, without it, the number of deaths from COVID-19 would likely have increased, similar to the Peruvian population.

In the instance of COVID-19 hospitalizations, doctors had a 72.0% higher risk than other healthcare workers, whereas technicians and health assistants had a 10.7% higher risk of hospitalization. The fact that technicians and health assistants have the highest risk of mortality, but a modestly increased chance of hospitalization may indicate inequity in access to hospitals, which may also explain the greater risk of COVID-19-related deaths in this cohort. It is possible that the efforts of the professional associations in obtaining air transport for their members and the coordination for their referrals, as is the case of the Peruvian Medical Association, have contributed to the timely hospitalization of its members, reducing their mortality [35]. Unfortunately, there is no school, society, or association of technicians and health assistants support in Peru that would ensure the timely hospitalization of its members, which may have put them at a disadvantage with other occupations that do have professional associations. Although it is true that our bibliographic review has not found studies that show inequities in access to hospitalization services in occupational groups of healthcare workers, this is possible since there is evidence of inequities for hospitalization in more disadvantaged or invisible groups during the SARS-CoV-2 pandemic [36, 37].

Probable reinfection was documented in 1.7% of healthcare workers. It was observed that the risk of probable reinfection was similar in the cohorts of healthcare workers studied; however, other occupational factors were relevant. The greatest risk of probable reinfection was found in those who worked outside the capital, particularly in establishments in the Amazon and the Andean region; a higher risk was also documented in those who had direct contact with COVID-19 cases in their workplace. It is possible that the greater limitations existing in the establishments of the MINSA/GORE, PNP/FAA, and outside the capital of Peru have contributed to the reinfection of healthcare workers during the pandemic’s greatest activity, moments in which there have been documented deficit of personal protective equipment, as well as greater exposure to COVID-19 due to the overwhelming patient demand [17, 34, 38–40].

Our study was conducted utilizing secondary sources, so it is probable that there are quality issues with data and some degree underreporting of adverse outcomes of SARS-CoV-2 infection; nevertheless, the fact that we considered many sources of information, as well as the verification and investigation of deaths, somewhat compensates for these limitations. Similarly, the data utilized could not identify which healthcare staff provided in-person care and which worked remotely nor measure the impact of the personal protective equipment deficit on infected healthcare worker hospitalization and death. Because the identification of SARS-CoV-2 lineages is not routinely performed in all cases of infection in Peru, it was not possible to confirm the reinfection of healthcare workers; therefore, probable reinfection was investigated. Lastly, our study did not have a control group made up of healthcare workers not infected by SARS-CoV-2 to compare our results, since, aside from the SINADEF base, the other databases used only provided data of infected people.

Despite these constraints, we believe that the acquired results are similar and comparable to what was observed in Peruvian healthcare workers during the pandemic.

## Conclusion

Peruvian technicians and health assistants would have a higher risk of death from COVID-19 than other healthcare workers, while doctors have a higher incidence of death probably linked to the high frequency of non-occupational risk factors such as age and the diagnosis of comorbidities. Doctors present a higher risk of hospitalization independent of comorbidities and age; likewise, all occupations show a similar risk of probable reinfection.

## Data Availability

Data may be made available by contacting the corresponding author.

## References

[1] International Labor Organization, World Health Organization. COVID-19: Occupational health and safety for healthcare workers. Geneva; QUIEN; 2021. https://www.who.int/publications/i/item/WHO-2019-nCoV-HCW_advice-2021.1.

[2] Urrea Vega EA, Antoniolli L, Teixeira Macedo AB, Gediel Pinheiro JM, Martins Dornelles T, Cócaro de Souza SB (2021). Risks of occupational illnesses among healthcare workers providing care to patients with COVID-19: an integrative review. Rev Lat Am Enfermagem.

[3] Fisher D, Heymann D (2020). Q&A: the novel coronavirus outbreak causing COVID-19. BMC Med.

[4] Firew T, Sano ED, Lee JW, Flores S, Lang K, Salman K (2020). Protecting the front line: a cross-sectional survey analysis of the occupational factors contributing to healthcare workers’ infection and psychological distress during the COVID-19 pandemic in the USA. BMJOpen..

[5] Amit S, Beni SA, Biber A, Grinberg A, Leshem E, Regev-Yochay G (2021). Postvaccination COVID-19 among Healthcare Workers, Israel. Emerg Infect Dis.

[6] Organization PAH (2021). Epidemiological Update: coronavirus Disease (COVID-19).

[7] Chang D, Xu H, Rebaza A, Sharma L, Dela Cruz CS (2020). Protecting health-care workers from subclinical coronavirus infection. Lancet Respir Med.

[8] Wu Z, McGoogan JM (2020). Characteristics of and Important Lessons From the Coronavirus Disease 2019 (COVID-19) Outbreak in China: summary of a Report of 72,314 Cases From the Chinese Center for Disease Control and Prevention. JAMA.

[9] National Liaison Center, Carlos III Health Institute. Report on the situation of COVID-19 in health personnel in Spain. Madrid: RENAVE. CNE. CNM (ISCIII); 2020.

[10] Gabriel M-A, Sanz-Gallen P, Arimany-Manso J (2020). Medicolegal assessment of COVID-19 infection in the workplace. Rev Esp Med Legal.

[11] Al Bujayr AA, Aljohar BA, Bin Saleh GM, Alanazi KH, Assiri AM (2021). Incidence and epidemiological characteristics of COVID-19 among health care workers in Saudi Arabia: a retrospective cohort study. J Infect Public Health.

[12] World Health Organization. COVID-19: Occupational health and safety for healthcare workers. Geneva: WHO; 2021. https://www.who.int/publications/i/item/WHO-2019-nCoV-HCW_advice-2021.1.

[13] Silva JS, Batista de Carvalho AR, Leite HC, Oliveira EN. Reflections on occupational risks in healthcare workers in times of the COVID-19 pandemic. Cuba Rev. sick 2020; 36(2). Available at: http://www.revenfermeria.sld.cu/index.php/enf/article/view/3738.

[14] World Health Organization (2021). Coronavirus disease (COVID-19): Accessibility and allocation of vaccines.

[15] Johns Hopkins University & Medicine. Mortality Analyzes: Mortality in the most affected countries. Baltimore: Johns Hopkins University; August 2020 (Accessed February 02, 2021). Available at: https://coronavirus.jhu.edu/data/mortality.

[16] Raraz-Vidal JG, Allpas-Gomez HL, Torres-Salome FK, Cabrera-Patiño WM, Alcántara-Leyva LM, Ramos-Gómez RP (2021). Work conditions and personal protective equipment against COVID-19 in health personnel, Lima-Peru. Rev Fac Med Hum.

[17] Neyra-Leon J, Huancahuari-Nuñez J, Diaz-Monge JC, Pinto JA (2020). The impact of COVID-19 on the healthcare workforce in Peru. J Public Health Policy.

[18] Padilla Machaca PM, Cárdenas Ramírez BE, Cabrera Cabrejos MC (2020). Impact of COVID-19 on liver diseases and public health in Peru. Rev Gastroenterol Peru.

[19] Government of Peru. Emergency Decree No. 026-2020 that establishes various exceptional and temporary measures to prevent the spread of the coronavirus (COVID-19) in the national territory [Extraordinary Edition March 15]. Lima: Official newspaper El Peruano; 2020, pp 1. Available at: https://busquedas.elperuano.pe/normaslegales/decreto-de-urgencia-que-establece-diversas-medidas-excepcion-decreto-de-urgencia-n-026-2020-1864948-1/.

[20] National Center for Epidemiology, Disease Prevention and Control, Ministry of Health of Peru. Sanitary Directive for Epidemiological Surveillance of coronavirus disease (COVID-19) in Peru [DS No. 135-MINSA/CDC-2021]. Lima: CDC/MINSA; 2001. Available at: https://www.gob.pe/institucion/minsa/normas-legales/2027213-881-2021-minsa.

[21] Gholami M, Fawad I, Shadan S, Rowaiee R, Ghanem H, Hassan Khamis A (2021). COVID-19 and healthcare workers: a systematic review and meta-analysis. Int J Infect Dis.

[22] Gómez-Ochoa SA, Franco OH, Rojas LZ, Raguindin PF, Roa-Díaz ZM, Wyssmann BM (2021). COVID-19 in health-care workers: a living systematic review and meta-analysis of prevalence, risk factors, clinical characteristics, and outcomes. Am J Epidemiol.

[23] Bandyopadhyay S, Baticulon RE, Kadhum M, Alser M, Ojuka DK, Badereddin Y (2020). Infection and mortality of healthcare workers worldwide from COVID-19: a systematic review. BMJ Glob Health.

[24] McNamara CL, McKee M, Stuckler D (2021). Precarious employment and health in the context of COVID-19: a rapid scoping umbrella review. Eur J Public Health.

[25] Llop-Gironés A, Vračar A, Llop-Gironés G, Benach J, Angeli-Silva L, Jaimez L (2021). Employment and working conditions of nurses: where and how health inequalities have increased during the COVID-19 pandemic?. Hum Resource Health.

[26] Pérez-Raya F, Cobos-Serrano JL, Ayuso-Murillo D, Fernández-Fernández P, Rodríguez-Gómez JA, Almeida SA (2021). COVID-19 impact on nurses in Spain: a considered opinion survey. Int Nurs Rev.

[27] Government of Peru. Supreme Decree No. 004-2012-SA that approves the Regulation of Law No. 28561, Law that regulates the work of health care technicians and assistants. Official Gazette El Peruano 2012; page 463365. Available at: https://busquedas.elperuano.pe/normaslegales/aproban-reglamento-de-la-ley-n-28561-ley-que-regula-el-tr-decreto-supremo-n-004-2012-sa-771039-2/.

[28] Faíco-Filho KS, Passarelli VC, Bellei N (2020). Is higher viral load in SARS-CoV-2 associated with death?. Am J Trop Med Hyg.

[29] McEllistrem MC, Clancy CJ, Buehrle DJ, Singh N, Lucas A, Sirianni V (2021). SARS-CoV-2 is associated with high viral loads in asymptomatic and recently symptomatic healthcare workers. PLoS ONE.

[30] Knudtzen FC, Jensen TG, Lindvig SO, Rasmussen LD, Madsen LW, Hoegh SV (2021). SARS-CoV-2 viral load as a predictor for disease severity in outpatients and hospitalized patients with COVID-19: a prospective cohort study. PLoS ONE.

[31] Robles-Pérez E, González-Díaz B, Miranda-García M, Borja-Aburto VH (2021). Infection and death by COVID-19 in a cohort of healthcare workers in Mexico. Scand J Work Environ Health.

[32] Nienhaus A, Hod R (2020). COVID-19 among Healthcare workers in Germany and Malaysia. Int J Environ Res Public Health.

[33] Ferland L, Carvalho C, Gomes Dias J, Lamb F, Adlhoch C, Suetens C (2022). Risk of hospitalization and death for healthcare workers with COVID-19 in nine European countries, January 2020-January 2021. J Hosp Infect.

[34] Escobar-Agreda S, Silva-Valencia J, Rojas-Mezarina L, Vargas-Herrera J (2021). Survival of healthcare workers infected with SARS-CoV-2 in the context of vaccination against COVID-19 in Peru. An Fac med.

[35] Galán-Rodas E, Tarazona-Fernández A, Palacios-Celi M (2020). Risk and death of doctors 100 days after the state of emergency due to COVID-19 in Peru. Acta Med Peru.

[36] Khanijahani A, Iezadi S, Gholipour K, Azami- Aghdash S, Naghibi D (2021). A systematic review of racial/ethnic and socioeconomic disparities in COVID-19. Int J Equity Health.

[37] Baksh RA, Pape SE, Smith J, Strydom A (2021). Understanding inequalities in COVID-19 outcomes following hospital admission for people with intellectual disability compared to the general population: a matched cohort study in the UK. BMJ Open.

[38] Valenzuela-Rodriguez G, Zambrano LI, Muñoz-Lara F, Pecho-Silva S, Arteaga-Livias K, Rodriguez-Morales AJ (2020). Intranational differences in the case fatality rates for COVID-19 among Peruvian physicians. Int J Infect Dis.

[39] Ramos W, Arrasco J, De La Cruz-Vargas JA, Ordóñez L, Vargas M, Seclén-Ubillús Y, et al. Epidemiological characteristics of deaths from COVID-19 in Peru during the Initial pandemic response. Healthcare 2022;10(12):2404. 10.3390/healthcare10122404 PMC9777767 36553928

[40] Pampa-Espinoza L, García M, Gavilán RG, Donaires L, Cabezas C, Rojas N (2021). First case of reinfection confirmed by SARS-CoV-2 in Peru. Rev Peru Med Exp Public Health.

